# Comparing mental health levels between caregivers of patients with severe mental disorders and caregivers of patients with rare genetic diseases

**DOI:** 10.1192/j.eurpsy.2025.694

**Published:** 2025-08-26

**Authors:** M. Di Vincenzo, F. Guida, S. Calamaro, R. Murolo, B. Prota, M. Luciano, G. Limongelli, G. Sampogna, A. Fiorillo

**Affiliations:** 1 Department of Psychiatry; 2 Inherited and Rare Cardiovascular Disease Unit, Department of Translational Medical Sciences, University of Campania “Luigi Vanvitelli”, Naples, Italy

## Abstract

**Introduction:**

Severe mental disorders and rare genetic diseases are chronic and highly disabling conditions requiring continuous assistance by caregivers, whose personal and social burden may result in mental health problems.

**Objectives:**

The current study aims to compare objective and subjective burden as well as levels of general well-being, anxiety, depressive symptoms, PTSD symptoms, sleep quality and suicidality between caregivers of patients with severe mental disorders, caregivers of patients aged 0-18 with rare genetic diseases, and caregivers of patients aged 50-85 with rare genetic diseases.

**Methods:**

Caregivers of patients with severe mental disorders were recruited at the Department of Psychiatry of University of Campania, Naples, if they were more than 18 and released consent. Caregivers of all patients with rare genetic diseases were recruited at the Inherited and Rare Cardiovascular Disease Unit of University of Campania, Naples, if they were more than 18 and released consent. Caregivers’ levels of personal and social burden, anxiety, depressive symptoms, PTSD symptoms, sleep quality and suicidality were assessed through standardized tools and compared between groups by carrying out analyses of variance.

**Results:**

Seventy-seven caregivers were included, mostly women (74.0%) with a mean age of 52.2±12.5 years. Caregivers of patients with severe mental disorders were mainly mothers (31.8%) or partners (31.8%) of patients, showing the highest levels of subjective burden, as well as avoidance and need to be informed about the illness adopted as problem-solving strategies. Caregivers of patients aged 0-18 with rare genetic diseases were mainly mothers of patients (76.5%), reporting the highest levels of received support and PTSD symptoms. Caregivers of patients aged 50-85 were usually partners of patients (52.4%), who showed the lowest levels of need to be informed about the illness and PTSD symptoms.

**Conclusions:**

Caregivers of patients suffering from chronic and disabling diseases such as severe mental disorders and rare genetic diseases are prone to develop mental health problems due to the persistent exposure to high levels of personal and social burden. To this extent, family psychosocial interventions may be effective strategies to be implemented in order to relieve the levels of burden, by taking into account the features of patient’s disease.

**Disclosure of Interest:**

None Declared

